# Hepatocellular-Targeted mRNA Delivery Using Functionalized Selenium Nanoparticles In Vitro

**DOI:** 10.3390/pharmaceutics13030298

**Published:** 2021-02-24

**Authors:** Dhireshan Singh, Moganavelli Singh

**Affiliations:** Nano-Gene and Drug Delivery Group, Discipline of Biochemistry, University of KwaZulu-Natal, Private Bag, Durban X54001, South Africa; 213554859@stu.ukzn.ac.za

**Keywords:** selenium nanoparticles, F*Luc*-mRNA, lactobionic acid, chitosan, targeting, gene expression, hepatocellular carcinoma

## Abstract

Selenium’s (Se) chemopreventative and therapeutic properties have attracted attention in nanomedicine. Se nanoparticles (SeNPs) retain these properties of Se while possessing lower toxicity and higher bioavailability, potentiating their use in gene delivery. This study aimed to formulate SeNPs for efficient binding and targeted delivery of F*Luc*-mRNA to hepatocellular carcinoma cells (HepG2) in vitro. The colorectal adenocarcinoma (Caco-2) and normal human embryonic kidney (HEK293) cells that do not have the asialoorosomucoid receptor (ASGPR) were utilized for comparison. SeNPs were functionalized with chitosan (CS), polyethylene glycol (PEG), and lactobionic acid (LA) for ASGPR targeting on HepG2 cells. Nanoparticles (NPs) and their mRNA-nanocomplexes were characterized by Fourier transform infra-red (FTIR) and UV-vis spectroscopy, transmission electron microscopy (TEM), and nanoparticle tracking analysis (NTA). Gel and fluorescence-based assays assessed the NP’s ability to bind and protect F*Luc*-mRNA. Cytotoxicity was determined using the -(4,5-dimethythiazol-2-yl)-2,5-diphenyl tetrazolium bromide (MTT) assay, while transgene expression was evaluated using the luciferase reporter gene assay. All NPs appeared spherical with sizes ranging 57.2–130.0 nm and zeta potentials 14.9–31.4 mV. NPs bound, compacted, and protected the mRNA from nuclease digestion and showed negligible cytotoxicity in vitro. Targeted gene expression was highest in the HepG2 cells using the LA targeted NPs. These NPs portend to be efficient nanocarriers of nucleic acids and warrant further investigation.

## 1. Introduction

The liver is one of the most vital organs of the body. It is responsible for controlling, regulating, and maintaining homeostasis in a plethora of biological functions and processes [1]. This, coupled with its close association with digested products and its ability to inactivate toxins, makes the liver vulnerable to numerous pathologies [1,2]. This can lead the liver to a state where operative surgery to correct its diseased state is unfavorable [3].

Liver cancer is the fifth most common cancer and the second leading cause of all cancer-related deaths worldwide [4]. From the many forms of liver cancer that originate from its various functional units and associated cells [5,6,7], hepatocellular carcinoma (HCC), which originates from the hepatocytes of the liver, is the most common and accounts for approximately 90% of all diagnosed cases [7,8]. The etiology of HCC is vast and diverse but still relatively unknown, occurring sporadically, through inheritance or continuous exposure to carcinogens [4,6,7,9]. The current treatment for HCC is dependent on early prognosis and pathology of the tumor, with surgical resection, liver transplantation followed by chemotherapy. However, the side effects and limitations of current treatments, such as lack of donors and multidrug resistance [10], render these current treatments powerless. Approximately 80% of reported HCC patients succumb to death within 6–8 months of initial diagnoses [11], necessitating the development of novel strategies for treating HCC.

Selenium (Se) is an essential nutritional element with a multifunctional role within the body. All organisms require Se to maintain and regulate proper biological functions [12,13]. Selenium compounds have often been used as a nutritional compound to promote animal and human health [14,15]. Increased Se supplementation has been correlated to a lower incidence of cardiovascular diseases, osteoarthritis, type 2 diabetes, antimicrobial activity, and neurologic disorders such as Alzheimer’s [14,16,17]. Selenium supplementation has also been shown to prevent the onset of hepatopathies that may lead to chronic injury to the liver and development of cirrhosis, which may later develop into HCC [18]. The use of Se as a chemotherapeutic agent (often in combination with drugs) in hepatic, lung, brain, breast, prostate, colon, and skin carcinomas, both in vitro and in vivo, have been documented [12,13,14]. The anticancer activity of Se has been linked to several mechanisms, including apoptosis; cell cycle arrest; redox regulation through pro-oxidant or antioxidant pathways; detoxification of cancer-inducing agents, such as binding cancer-causing metals; increased stimulation of the immune system; and angiogenesis inhibition [19,20]. However, the medical use of Se is somewhat hindered due to the dose-dependent nature of the element; Se shows a “U”-shaped dose effectiveness with moderately high levels of Se, close to the toxicity limit, capable of inducing anticancer activity [13,14,16,20]. This may be a limitation of Se in medical applications due to the abundance of Se compounds that can be obtained from the diet, water, or from the air [14]. SeNPs have thus been employed therapeutically to combat pathologies such as cancer, microbial infection, and diabetes due to their anti-inflammatory, antioxidative, anticancer, wound healing ability, drug and metal-binding activity, and diagnostic capability in the form of imaging and gene and drug nanocarrier ability [12,21,22].

Nanotherapeutics offers an advantage over the conventional therapies of today with Se nanoparticles (SeNPs) providing a novel strategy in medical applications. Various studies have reported SeNPs to possess lower toxicity, higher bioavailability, biodegradability, increased biological activity for inducing selenoproteins, the ability to scavenge free radicals, antioxidant activity preventing oxidative DNA, and cancer prevention compared to their inorganic or organic Se counterparts [13,21,22] while still retaining the beneficial advantages associated with the inorganic and organic Se species. SeNPs may have found a niche in therapeutic gene and drug delivery owing to their favorable properties mentioned above, in addition to the possibility that they may act synergistically with their therapeutic cargo, which in this study, is the mRNA. This potential is yet to be fully explored.

Since cancer is fundamentally a genetic disease, the use of gene therapy offers a novel and promising approach to treat an array of liver diseases, from cancer to fibrosis and cirrhosis [23,24,25]. Gene therapy, with the use of nanoscale gene delivery vehicles, can provide promising targeted treatment strategies that can reduce adverse side effects [24,26]. RNA-based therapies have shown precedence over DNA-based therapeutics, exhibiting high levels of transient gene expression and low chances of insertion into the host genome [24,27]. Specifically, mRNA therapy has demonstrated significant advancement in vaccine development, immune-oncology, and protein replacement [28,29]. However, its short half-life, inherent instability, and rapid degradation by RNases make it a challenging perspective to pursue [30,31]. Although modifications to the mRNA strand can reduce the inherent instability, degradation by RNases en route to the target cell still poses a major challenge. Thus, mediation of RNA often requires the use of a vector [31,32,33]. Chitosan SeNPs (Cs-SeNPs) have been explored previously for the delivery of siRNA [34] and mRNA [35], and dendrimer-SeNPs were recently used for the delivery of a luciferase reporter gene, pCMV*-luc-*DNA [36]. This study explores the novel use of SeNPs as liver-targeted mRNA-based nanocarriers and evaluated their luciferase gene expression as a proof of principle study.

## 2. Materials and Methods

### 2.1. Materials

The F*luc*-mRNA (sequence in Supplementary Figure S1), modified with 5*-* methylcytidine and pseudouridine, was supplied by TriLink BioTechnologies, Inc (San Diego, CA, USA). Dialysis tubing (MWCO = 12 kDa), sodium selenite, ascorbic acid, chitosan >75% deacetylated, polyethylene glycol -2-amino ethyl ether acetic acid, *N*-ethyl-*N*′-(3-dimethyl aminopropyl) carbodiimide (EDC), *N*-hydroxysuccinimide (NHS), lactobionic acid (LA), sodium hydroxide, and bicinchoninic acid (BCA) were purchased from Sigma-Aldrich Chemical Co., (St Louis, MO, USA). Acetic acid, 2-[4-(2-hydroxyethyl)-1-piperazinyl] ethane sulphonic acid (HEPES), 3-(4,5-dimethythiazol-2-yl)-2,5-diphenyl tetrazolium bromide (MTT), tris (hydroxymethyl) aminomethane, sodium dihydrogen phosphate, ethylenediaminetetraacetic acid (EDTA) disodium salt, dimethyl sulfoxide (DMSO), phosphate-buffered saline tablets [PBS, (140 mM NaCl, 10 mM phosphate buffer, 3 mM KCl)], and ethidium bromide were purchased from Merck (Darmstadt, Germany). Ultrapure grade agarose was purchased from Bio-Rad Laboratories, Inc. (Richmond, VA, USA). Human embryonic kidney (HEK293), colorectal adenocarcinoma (Caco-2), and hepatocellular carcinoma (HepG2) human cell lines were originally sourced from the ATCC (Pty) Ltd., (Manassas, VA, USA). Eagle’s Minimum Essential Medium (EMEM), L-glutamine (4.5 g/mL), trypsin-EDTA mixture (0.25% *w*/*v* trypsin, 0.1% *w*/*v* EDTA), and antibiotic (Penicillin (5000 units/mL)/Streptomycin (5000 µg/mL)) were purchased from Lonza BioWhittaker (Verviers, Belgium). Fetal bovine serum (FBS) was purchased from Gibco Invitrogen (Karlsruhe, Germany). The luciferase assay kit, reagent (20 mM tricine, 1.1 mM magnesium carbonate hydroxide, pentahydrate, 2.7 mM magnesium sulphate, 0.1 mM EDTA, 33.3 mM dithiothreitol, 270 μM coenzyme A, 470 μM luciferin, and 530 μM ATP), and 5× lysis buffer (25 mM trisphosphate, pH 7.8; 2 mM dithiothreitol, 2 mM 1,2-diaminocyclohexane–N,N,N′,N′-tetraacetic acid, 10% (*v*/*v*) Glycerol, and 1% (*v*/*v*) Triton X-100) were supplied by Promega Corporation (Madison, USA). All sterile consumable plasticware for tissue culture was obtained from Corning Incorporated (New York, NY, USA). All chemicals were of analytical grade, and ultrapure 18 Mohm (MilliQ, Millipore Sigma, Burlington, MA, USA) water was used in all preparations.

### 2.2. SeNP Synthesis and Functionalization

#### 2.2.1. Synthesis of SeNPs

**Figure 1:**
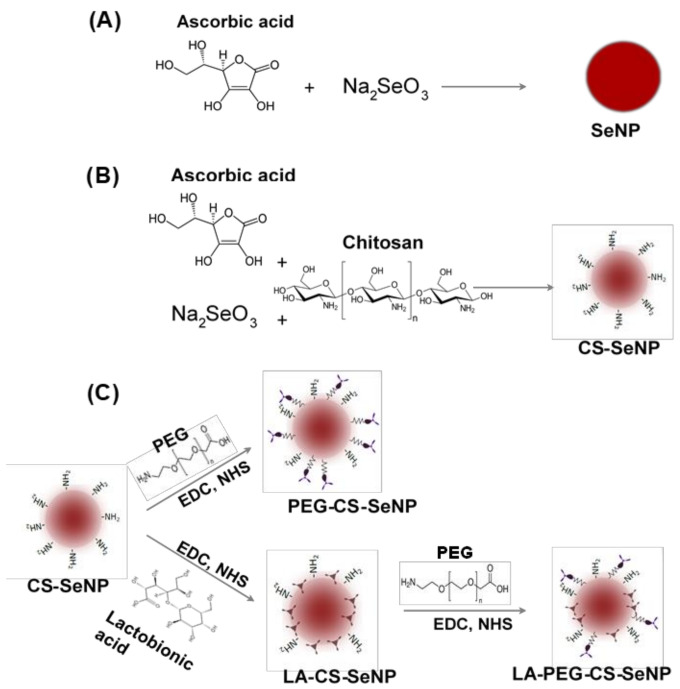
Scheme for the synthesis of (**A**) Selenium nanoparticles (SeNPs), (**B**) chitosan-selenium nanoparticles (CS-SeNPs), and (**C**) lactobionic acid-chitosan-selenium nanoparticles (LA-CS-SeNPs), polyethylene glycol-chitosan-selenium nanoparticles (PEG-CS-SeNPs) and lactobionic acid-polyethylene glycol-chitosan-selenium nanoparticles (LA-PEG-CS-SeNPs).

The SeNPs were synthesized (Figure 1A) chemically by the reduction of sodium selenite (Na_2_SeO_3_) with ascorbic acid, as described previously with slight modifications [34,35,36]. Briefly, 0.2 mL of a 0.51 M Na_2_SeO_3_ solution was added dropwise with stirring to 7.5 mL of a freshly prepared 0.23 M ascorbic acid solution and left to stir for a further 30 min. A color change from colorless to orange was noted, indicating the formation of SeNPs. The SeNP solution was then dialyzed (12 kDa MWCO) against MilliQ water at room temperature (25 °C) for 24 h to remove any unreacted material. NPs were stored in 18 Mohm water.

#### 2.2.2. Preparation of CS Encapsulated Functionalized SeNPs

CS encapsulated SeNPs (CS-SeNPs) were synthesized (Figure 1B) as described previously [34,35] with slight modifications. Briefly, 0.1% (*w*/*v*) CS solution (15 mL in acetic acid) was added slowly to 7.5 mL of a 0.23 M ascorbic acid solution, followed by the addition of 0.2 mL of a 0.51 M Na_2_SeO_3_ solution under constant stirring for 30 min. A color change from colorless to a deep red indicated a positive reaction and formation of CS encapsulated SeNPs. Thereafter, the CS-SeNP solution was dialyzed and stored as in Section 2.2.1.

#### 2.2.3. Preparation of Lactobionic Acid-Chitosan (LA-CS-SeNPs) Functionalized SeNPs

LA-CS SeNP synthesis was adapted from [37] with slight modifications (Figure 1C). Approximately 1.46 mg (11% *w*/*w*) of LA was added to 10 mL (1.182 mg/mL) of the CS encapsulated SeNPs with constant stirring. Thereafter, 3.83 mg (1.763 mM) EDC and 2.30 mg (1.998 mM) NHS (1:1.3 molar ratio) were added to the LA-CS SeNP solution, which was stirred for 72 h at room temperature with the pH maintained between 4 and 6. Samples were then dialyzed and stored as in Section 2.2.1.

#### 2.2.4. Preparation of PEG-CS Functionalized SeNPs

SeNPs were PEGylated using polyethylene glycol-2-amino ethyl ether acetic acid (PEG) conjugated to the amino group on CS in the CS-SeNPs (Figure 1C). The method described in Section 2.2.3 was further adapted to PEGylate CS-SeNPs within an anaerobic chamber. The main anaerobic chamber was purged with argon gas, and pressure maintained at 1 atm. An anaerobic indicator strip (OXOID^™^) was used to detect the presence of O_2_ within the chamber. Approximately 3.83 mg of EDC and 2.30 mg of NHS were added to a separate reaction vessel. The CS-SeNP solution (10 mL) was purged with argon gas to remove any diluted O_2_, followed by the addition of 0.16 mg (~2.2% *w*/*w*) of PEG and the final addition of the EDC/NHS mixture. The PEG-CS-SeNP solution was then sealed and stirred for 72 h at room temperature at pH 4–6. Samples were dialyzed and stored as in Section 2.2.1.

#### 2.2.5. Preparation of PEG-LA-CS Functionalized SeNPs

The procedure conducted ensured that the carboxyl group of the LA did not bind to the amino group on the PEG promoting successful binding of the PEG to the amino group on the CS. Briefly, 0.16 mg PEG was added to 10 mL (1.325 mg/mL) LA-CS-SeNP solution (purged with argon) within an anaerobic chamber devoid of atmospheric gas. Thereafter, 3.83 mg EDC and 2.30 mg NHS were added to the PEG-LA-CS-SeNP solution (Figure 1C). The samples were then sealed and stirred for 72 h at room temperature, maintaining the pH between 4 and 6. Samples were then dialyzed and stored as in Section 2.2.1.

### 2.3. Formation of Nanocomplexes

Prior to handling, the F*luc*-mRNA (Supplementary Figure S1) in RNase free water was thawed on ice. Nanocomplexes were prepared in a class-II biohazard hood under sterile conditions. Prior to complexes being formed, all surfaces, reagents, and apparatus were sterilized using 70% ethanol, wiped down with a nuclease decontamination solution (100 mL bleach, 10 g sodium hydroxide, 10 g SDS, and 6.72 g sodium bicarbonate diluted in 1 L of 18 Mohm water) and PCR Clean™ (Minerva biolabs^®^), which degraded DNA and RNA contaminants. Nanocomplexes were prepared by the addition of varying concentrations of the CS-Se, LA-CS-Se, PEG-CS-Se, and PEG-LA-CS-Se NPs to a constant concentration (0.30 µg/µL) of F*luc*-mRNA, producing a range of (*w*/*w*) ratios of NP:mRNA. The nanocomplex solutions were brought up to a constant volume of 10 μL with HEPES buffered saline (HBS) (20 mM HEPES, 150 mM NaCl, pH 7.4). The nanocomplexes were incubated at room temperature for 45 min.

### 2.4. Characterization of Nanoparticles and Nanocomplexes

#### 2.4.1. UV-Visible Spectroscopy

Formation of SeNPs and their functionalized counterparts was confirmed using UV spectroscopy, as evidenced by a shift in the wavelength peak of the different SeNPs. Briefly, the absorbance maximum of all NPs (10 µL) was measured between 200 and 800 nm at 2 nm intervals at a read speed of 400 nm/min on a Jasco V-730 UV-visible/ NIR Bio spectrophotometer (Hachioji, Japan).

#### 2.4.2. Fourier-Transform Infrared Spectroscopy (FTIR)

Fourier-transform infrared spectroscopy was employed to identify the presence of enhancing motifs on functionalized SeNPs and to confirm the binding of LA and PEG to the amino group of CS through the visualization of specific functional group peaks. FTIR was conducted using a Perkin Elmer Spectrum 100 FTIR spectrometer with a universal ATR sampling accessory scanning from 4000 to 380 cm^−1^.

#### 2.4.3. Nanoparticle Tracking Analyses (NTA)

Hydrodynamic size and zeta potentials of all NPs and nanocomplexes at optimal binding ratios were determined using nanoparticle tracking analyses (NTA). Nanocomplexes were freshly prepared. All samples were diluted 1:1000 in 18 Mohm water and zeta potentials and hydrodynamic sizes measured in a Nanosight NS-500 (Malvern Instruments, Worcestershire, UK) at 25 °C. NTA software v3.0 (Malvern Instruments, Worcestershire, UK, 2014) was used to calculate the accurate hydrodynamic diameters using the Stokes–Einstein equation and the zeta potential using Smoluchowski approximation.

#### 2.4.4. Transmission Electron Microscopy (TEM)

Morphological and size distribution of the NPs and nanocomplexes were determined by TEM. NPs were sonicated before being added to individual 400-mesh carbon-coated copper grids (Ted Pella Inc. Redding, CA, USA) and allowed to dry at room temperature for 30 min. The grids were then loaded onto the sample holder, and sample images were viewed in a JOEL JEM-1010 (Jeol, Tokyo, Japan) transmission electron microscope. Images were captured using iTEM Soft Imaging Systems (SIS) Megaview III fitted with a side-mounted digital camera (3-megapixels) and analyzed using analySIS LS Research v2.6 (Olympus Soft Imaging Solutions GmbH, Münster, Germany, 2004) to calculate the average particle diameter for each NP.

### 2.5. Band Shift Assay

A band shift assay [38] was conducted to determine the amount of functionalized SeNPs required to bind and condense 0.3 µg/µL mRNA completely. Nanocomplexes were formed as in Section 2.3. Following incubation, 3 µL of gel loading buffer (glycerol 50%, bromophenol blue 0.05%, and xylene cyanol 0.05%, Merck, Darmstadt, Germany) was added to each nanocomplex solution. A control containing only 0.3 µg mRNA was used to assess normal migration of mRNA not bound to the NPs. Electrophoresis was conducted on a 2% agarose gel containing ethidium bromide in TBE buffer for 30 min at 55 V. Gels were viewed in a Syngene G-box imaging system under 300 nm transillumination and images captured using GeneSnap software (version 1, 2011, Cambridge, UK). The sub-optimal and supra-optimal binding ratios below and above the optimal ratio were utilized in further studies (Supplementary Table S1).

### 2.6. Ethidium Bromide Intercalation Assay

An ethidium bromide intercalation assay [38] determined the ability of functionalized SeNPs (FSeNPs) to condense and compact mRNA via electrostatic interaction. Briefly, 2 µL of ethidium bromide (EB) (50 µg/µL) was added to a black 96-well plate together with 100 µL of HBS. This was taken as the baseline (0%) fluorescence. Fluorescence was measured at an excitation and emission wavelength of 525 nm and 580 nm, respectively. Thereafter, 1 µL of mRNA (0.3 µg) was added to the respective wells and incubated at room temperature for 10 min to allow for the EB to intercalate with the mRNA. This fluorescence measurement was taken as 100% fluorescence. Thereafter, 1 µL aliquots of the respective FSeNPs were added into each well. Samples were thoroughly mixed, and fluorescence measured using a GloMax^®^-Multi Detection System (Promega BioSystems, Sunnyvale, CA, USA) until a plateau in fluorescence was reached. The relative fluorescence was then plotted against the amount of FSeNPs using the following Equation (1):

(1) $$F_{r}\left(\%\right)\;=\;\frac{F_{i}\;{-\;F}_{0}}{F_{\max}\;{-\;F}_{0}}\;\times \;100$$

where, *F_r_* is the relative fluorescence of the sample normalized against the HBS-EB fluorescence, *F*_0_ is the fluorescence HBS-EB mixture in the absence of mRNA, *F_max_* is the fluorescence of the HBS-EB mixture after addition of mRNA and in the absence of FSeNPs, and *F_i_* is the fluorescence of the sample at a given concentration.

### 2.7. Nuclease Protection Assay

A nuclease protection assay [38] was conducted to assess the extent to which the FSeNPs were able to protect mRNA from nuclease digestion. FSeNP:mRNA nanocomplexes were prepared as in Section 2.3 but using the optimal, sub-optimal, and supra-optimal ratios (Supplementary Table S1) obtained from Section 2.5. Following nanocomplex formation, 10% (*v*/*v*) fetal bovine serum (FBS) was added to the respective nanocomplexes. Two controls, a positive control containing only mRNA with no FBS and a negative control containing mRNA treated with FBS, were included. All samples were incubated at 37 °C for 4 h. Thereafter, 10 mM of EDTA was added to each sample to stop the nuclease action, followed by the addition of 0.5% (*w*/*v*) SDS to liberate the mRNA from the nanocomplex. The samples were then incubated for a further 20 min at 55 °C. Samples were then subjected to agarose gel electrophoresis as described as in Section 2.5.

### 2.8. Cell Viability Assay

The viability of the cells in the presence of nanocomplexes at the sub-optimal, optimal, and supra-optimal ratios (Supplementary Table S1) was assessed using the 3-(4,5-dimethythiazol-2-yl)-2,5-diphenyl tetrazolium bromide (MTT) assay. HEK293, Caco-2, and HepG2 cells were seeded at a density of 2–2.5 × 10^4^ cells/well into 96-well plates containing 100 µL EMEM (supplemented with L-glutamine (4.5 g/mL), 10% FBS, and 1% antibiotics) and incubated in a HEPA Class 100 Steri-Cult CO_2_ incubator (Thermo-Electron Corporation, Waltham, MA, USA) at 37 °C in 5% CO_2_ for 24 h to allow for attachment of the cells. The EMEM was then renewed and the cells treated with the respective nanocomplexes. A control containing untreated cells was also included. All assays were conducted in triplicate. Cells were incubated for 48 h at 37 °C, followed by replacement of the medium with 100 µL of fresh EMEM containing 10 µL of MTT reagent (5 mg/mL, in PBS) and incubation of the cells at 37 °C for a further 4 h. Thereafter, the medium containing the MTT reagent was decanted from the wells and 100 µL of DMSO added to each well to dissolve the metabolized formazan crystals. The absorbance was then measured in a Mindray MR-96A microplate reader (Vacutec, Hamburg, Germany) at 570 nm using DMSO as a blank with the average absorbance of the untreated cell control taken as 100% cell viability.

### 2.9. Apoptosis

The dual acridine orange/ethidium bromide (AO/EB) staining was utilized to determine if there was any apoptotic induction by the nanocomplexes at their optimal binding ratios. HEK293, Caco-2, and HepG2 cells were seeded at a concentration of 5–6 × 10^4^ cells/well into 48-well plates and incubated for 24 h at 37 °C as in Section 2.8. Thereafter, the medium (EMEM) was replenished and cells treated with the optimal nanocomplex, in keeping with the cell density used. A positive control containing untreated cells was included, and all tests were done in triplicate. Following incubation, the medium was removed, and cells were washed with 100 µL of PBS. Thereafter, 10 µL of AO/EB (100 mg/mL, AO:EB (1:1) in PBS)) solution was added to each well, and plates were gently shaken for 5 min at room temperature. Cells were then washed with PBS and fluorescence viewed using an Olympus fluorescence microscope fitted with a CC12 fluorescence camera (Olympus Co., Tokyo, Japan). The apoptotic indices were calculated using the following Equation (2):

(2) $$Apoptotic\;Index\;=\;\frac{Number\;of\;apoptotic\;cells}{Total\;number\;of\;cells\;counted}$$

### 2.10. Transfection Activity: Luciferase Reporter Gene Assay

The transfection efficiency of all nanocomplexes was assessed quantitatively using a luciferase reporter gene assay. All tests were done in triplicate and included two controls, a cell only control (untreated cells) and a mRNA control (cells treated with naked mRNA). Cells were seeded into 96-well plates at a concentration of 2–2.5 × 10^4^ cells/well and incubated at 37 °C for 24 h as in Section 2.8. The medium (EMEM) was then replaced with fresh medium, and cells were treated with the respective nanocomplexes and incubated at 37 °C for 48 h. Following incubation, the medium was removed, and cells were washed twice with 20 µL of PBS. Thereafter, 60 µL of a 1 × lysis buffer was added to each well, and cells were shaken for 15 min at 30 revs/min. Lysed cells were dislodged by scraping and pelleted at 12,000× *g* for 5 s. Approximately 20 µL of the cell-free supernatant was added to a white 96-well plate, followed by direct injection of 100 µL of the luciferase assay reagent into the wells. Luminescence was measured in a GloMax^®^-Multi Detection System (Promega Biosystems, Sunnyvale, CA, USA). The standard BCA assay was used to determine the protein concentrations of the cell-free supernatants. Standard BSA solutions (ranging from 0 to 30 μg/50 μL in increments of 5 μg/50 μL) were prepared in a final volume of 50 μL with ultrapure water. These were mixed with the BCA working reagent (BCA solution:copper (II) sulfate solution, 50:1 *v*/*v*; 1 mL) and maintained at 37 °C for 30 min. Solutions were cooled to room temperature, and absorbance was measured at 540 nm (Mindray microplate reader, MR 96A, Vacutec, Hamburg, Germany). This data was used to construct a protein standard curve. The cell-free supernatants (50 μL) were mixed with the BCA working reagent (1 mL) and incubated (37 °C, 30 min). The soluble protein concentration of cell lysates was obtained via extrapolation from the standard curve. The resulting luciferase activity was normalized against the protein concentration and expressed as RLU/mg protein.

### 2.11. Receptor Competition Assay

The efficiency of uptake of LA-CS-SeNPs and PEG-LA-CS-SeNPs by receptor-mediated endocytosis through recognition of the asialoorosomucoid receptor (ASGPR1) was assessed further using a competition binding assay. HepG2 cells that overexpressed the ASGPR1 were seeded and incubated as in Section 2.10. Following incubation for 24 h, free LA at a concentration of 1 mg/mL was added to flood the receptors on the cells prior to addition of the LA-CS-Se and PEG-LA-CS-Se nanocomplexes (prepared as previously). Cells were then incubated for 48 h and subjected to the luciferase assay described in Section 2.10.

### 2.12. Statistical Analysis

Multiple comparisons grouped two-way analyses of variants (ANOVA) was used to compare means for both MTT and Luciferase assays using the Tukeys test in the statistical software program GraphPad Prism version 6.01 (GraphPad Software, La Jolla, CA, USA). Data shown are presented as mean ± standard deviation (*n* = 3). Statistical significance of the *p*-value was set at * *p* < 0.05 or ** *p* < 0.01.

## 3. Results

For ease of reading, the following abbreviations will be used for each nanoparticle (NP): Chitosan encapsulated SeNPs (CS-SeNPs), lactobionic acid conjugated chitosan SeNPs (LA-CS SeNPs), polyethylene glycol conjugated chitosan encapsulated SeNP (PEG-CS SeNPs) and polyethylene glycol-lactobionic acid conjugated chitosan encapsulated SeNPs (PEG-LA-CS SeNPs). All these NPs are also collectively abbreviated as functionalized SeNPs (FSeNPs).

**Figure 2:**
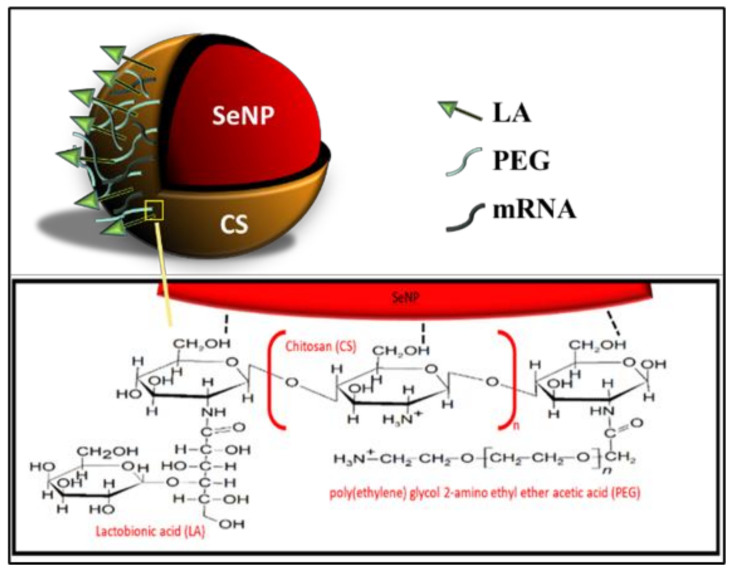
An illustration of the fully functionalized SeNP.

All NPs were successfully synthesized, with Figure 1 outlining the overall synthesis scheme and Figure 2 providing an illustration of the functionalized SeNP.

### 3.1. Visual Confirmation and UV-Visible Spectroscopy of SeNPs and FSeNPs

**Figure 3:**
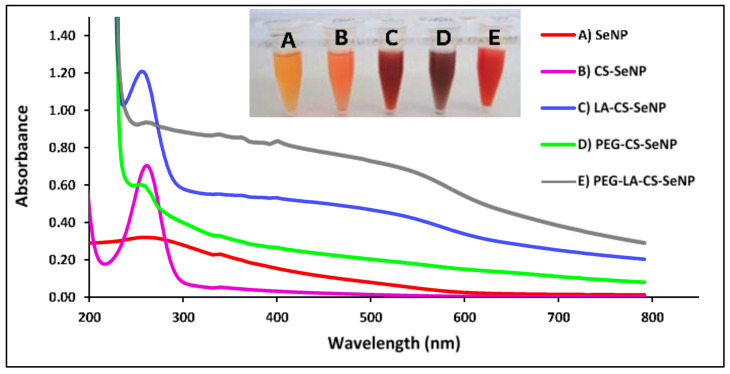
UV-visible spectra of (**A**) SeNPs, (**B**) CS-SeNPs, (**C**) LA-CS-SeNPs, (**D**) PEG-CS-SeNPs, and (**E**) PEG-LA-CS SeNPs and color changes (inset) observed during synthesis.

A color change from colorless to orange indicated the formation of the SeNPs (Figure 3A-inset), with CS encapsulation of the SeNPs producing an orange/red color (Figure 3B-inset) and a notable increase in the viscosity. The LA-CS-SeNP solution appeared brick red (Figure 3C-inset), the PEG-CS-SeNP appeared dark red (Figure 3D-inset), and the PEG-LA-CS-SeNP solution a bright red (Figure 3E-inset). The UV-vis profile of the NPs showed the characteristic SeNP peak between 200 and 300 nm. The **λ**_max_ for the SeNPs was 267.4 nm, while blue shifts in the **λ**_max_ were evident for CS-SeNPs, LA-CS-SeNPs, PEG-CS-SeNPs, and PEG-LA-CS-SeNPs to 266, 261, 261.2 and 262.8 nm, respectively (Figure 3).

### 3.2. Fourier Transform Infrared (FTIR) Spectroscopy

FTIR characterization further confirmed the successful synthesis and functionalization of the SeNPs. Peaks were assigned according to that in literature [39,40,41,42,43,44]. For CS-SeNPs (Supplementary Figure S1B), the peak at 3238 cm^−1^ was assigned to O−H and N–H stretching, while peaks at 2918, 1618, 1375 and 1032 cm^−1^ were assigned to C−H stretching, N–H bending, C–N stretching, and C–O stretching, respectively, [39,40]. For LA-CS-SeNP, and PEG-LA-CS-SeNPs (Supplementary Figure S2A,C), pyran ring peaks were detectable close to 900 cm^−1^ with peaks at 917 and 915 cm^−1^, respectively [41]. Peaks at 3347 and 3337 cm^−1^ were assigned to O−H stretching, peaks at 1652 and 1642 cm^−1^ corresponded to C=O stretching, peaks at 2913 and 2933 cm^−1^ corresponded to C–H stretching, with stretching of C–O groups seen at ~1074 cm^−1^ indicating the presence of LA [39,41].

For PEG-CS-SeNPs and PEG-LA-CS-SeNPs (Supplementary Figure S2B,C), peaks at 1158 and 1121 cm^−1^ corresponded to C−O−C stretching but showed a redshift (~1101 cm^−1^) [39]. This is an indication of the association between the oxygen in the C−O−C bond of PEG and Se atoms [42]. The C–H stretching of the CH_2_ groups of PEG was seen at 2855 cm^−1^ for the PEG-LA-CS-SeNPs [39,43] but was not evident for PEG-CS-SeNPs, although a shift in the O−H peak from 3272 to 3269 cm^−1^, as well as an increased broadness of the peak, was observed. This may indicate the presence of water molecules that may have masked the peaks, resulting in slight inconsistencies. The peaks at ~1600 and ~1500 cm^−1^ indicated the presence of amide bonding with C=O stretching and N–H bending within the amide bond [39]. Peaks at 1564, 1547, 1563 cm^−1^ in the LA-CS-SeNP, PEG-CS-SeNP and PEG-LA-CS-SeNP confirmed the bonding of the respective ligands to CS. Peaks at ~1700 cm^−1^, which correspond to the C=O stretch of free carboxylic acids, were not evident, indicating successful attachment of ligands [44].

### 3.3. Morphology, Size and Zeta Potential of SeNPs and FSeNPs

**Figure 4:**
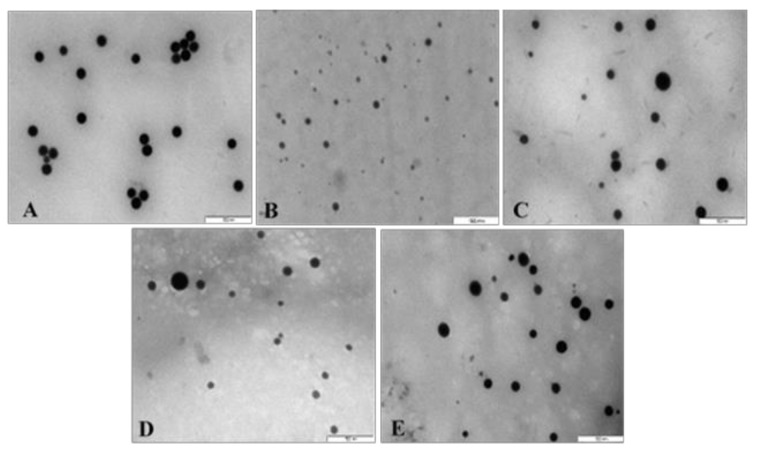
TEM images of (**A**) SeNPs, (**B**) CS-SeNPs, (**C**) LA-CS-SeNPs, (**D**) PEG-SeNPs, and (**E**) PEG-LA-CS-SeNPs. Scale = 500 nm.

All NPs appeared spherical under TEM with uniform distribution and no significant agglomeration. Visible size differences were also observed for the FSeNPs (Figure 4B–E) compared to the SeNPs (Figure 4A), with CS-SeNPs being the smallest (Figure 4B). The TEM based sizes of the NPs ranged from 80.52 nm to 123.49 nm (Table 1). From NTA analysis (Table 1), the hydrodynamic diameter of the NPs ranged from 57.2 nm to 130.0 nm with zeta potentials ranging from −45.8 to 31.4 mV. SeNPs (83.8 nm) were greatly reduced in size upon CS conjugation (57.2 nm) and with a change in zeta potential from −45.8 mV to +20.5 mV. However, LA and PEG conjugation produced an increase in the size of the LA-CS-SeNP and PEG-CS-SeNP to 130.0 and 91.4 nm, respectively. PEG-CS-SeNPs had a higher zeta potential (31.4 mV) indicating better colloidal stability compared to LA-CS-SeNPs (16.9 mV) and PEG-LA-CS-SeNP (14.9 mV).

### 3.4. Hydrodynamic Size and Zeta Potential of FSeNP: mRNA Nanocomplexes

The CS-Se and PEG-CS-Se nanocomplexes showed a significant decrease in their zeta potential to −10.4 and −22.5 mV with an increase in the hydrodynamic size to 70.6 and 110.2 nm, respectively (Table 2). In contrast, LA-CS-SeNPs and PEG-CS-SeNPs had a reduced zeta potential (−3.7 and −4.1 mV) with increases in their hydrodynamic sizes (176.0 and 263.9 nm). Overall, the polydispersity indices for the NPs and nanocomplexes were below 0.2, indicating they were monodisperse.

### 3.5. Band Shift Assay

**Figure 5:**
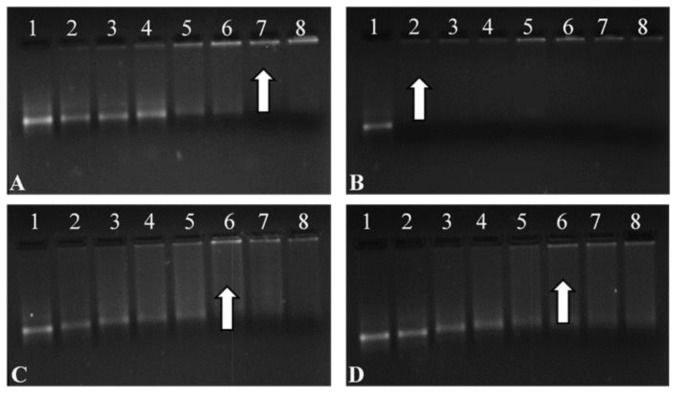
Band shift assay of the F*luc*-mRNA nanocomplexes with (**A**) CS-SeNPs, (**B**) LA-CS-SeNPs, (**C**) PEG-CS-SeNPs, and (**D**) LA-PEG-CS-SeNPs, using a 2% agarose gel. Arrows indicate optimal binding ratios. Lane 1: Control (naked mRNA (0.3 µg/µL)). Lanes 2–8 (**A**) mRNA:CS-SeNP *^w^/_w_* ratios (1:0.5; 1:1; 1:2; 1:3; 1:4; 1:5 and 1:5.5), (**B**) mRNA:LA-CS-SeNP *^w^/_w_* ratios (1:6; 1:7; 1:8; 1:9; 1:10, 1:11 and 1:12), (**C**) mRNA:PEG-CS-SeNP *^w^/_w_* ratios (1:1; 1:2; 1:3; 1:4; 1:5; 1:6 and 1:7), and mRNA PEG-LA-CS-SeNP *^w^/_w_* ratios (1:1; 1:2; 1:3; 1:4; 1:5; 1:6 and 1:7).

All FSeNPs showed complex mRNA and an innate ability to bind (Figure 5). The addition of varying amounts of FSeNPs was pursued until an electroneutral state was reached and the optimal ratio of mRNA:NP was identified. The naked control mRNA showed its natural integral bands (Lanes 1). Lanes 2–6, 2–5, and 2–5 (Figure 5A,C,D), show incomplete binding of mRNA. Migration bands seen in the gel indicated that the charges on the mRNA were not completely neutralized, resulting in migration of the mRNA into the gel.

Lanes 7–8, 2–8, 6–8 and 6–8 (Figure 5A–D) show binding of mRNA, depicted as a fluorescent band near the well. Optimum ratios (arrows) of the CS-SeNPs, LA-CS-SeNPs, PEG-CS-SeNPs, and LA-PEG-CS-SeNPs were identified at a mRNA:NP (*w*/*w*) ratio of 1:5, 1:6, 1:5, and 1:5, respectively. Sub-optimum and supra-optimum ratios were chosen 0.5 above and below these ratios and are represented in Supplementary Table S1 These ratios were utilized in further assays.

### 3.6. Ethidium Bromide Intercalation Assay

**Figure 6:**
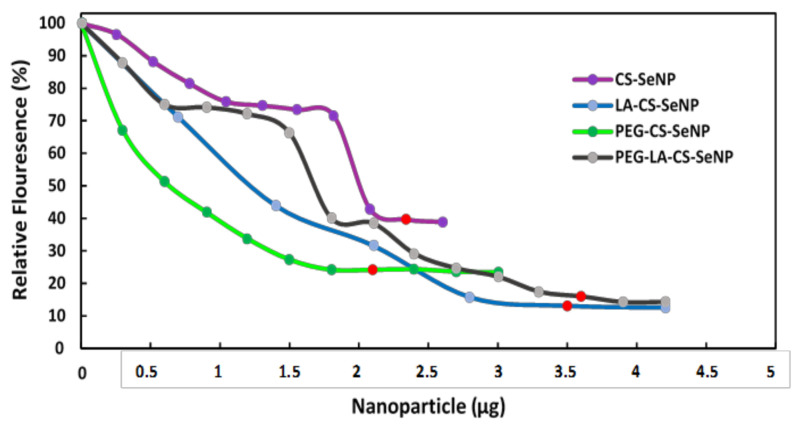
Ethidium bromide intercalation assay of CS-SeNPs, LA-CS-SeNPs, PEG-CS-SeNPs, and LA-PEG-CS-SeNPs. Red dots indicate maximum compaction of mRNA by the NPs.

All FSeNPs were capable of condensing mRNA to varying extents, as evidenced by a decrease in the fluorescence (Figure 6). As the NPs were added to the mRNA/EB mixture, the fluorescence decreased until a plateau was reached, at which point the maximum amount of mRNA was compacted by the individual NPs. CS-SeNPs showed the lowest compaction potential with a fluorescent decay to 39.6%, while the ligand modified NPs produced the highest compaction potential with fluorescence decays down to 13.1%, 24.2%, and 16.0% for the LA-CS-SeNPs, PEG-CS-SeNPs, and PEG-LA-CS-SeNPs, respectively.

### 3.7. Nuclease Protection Assay

**Figure 7:**
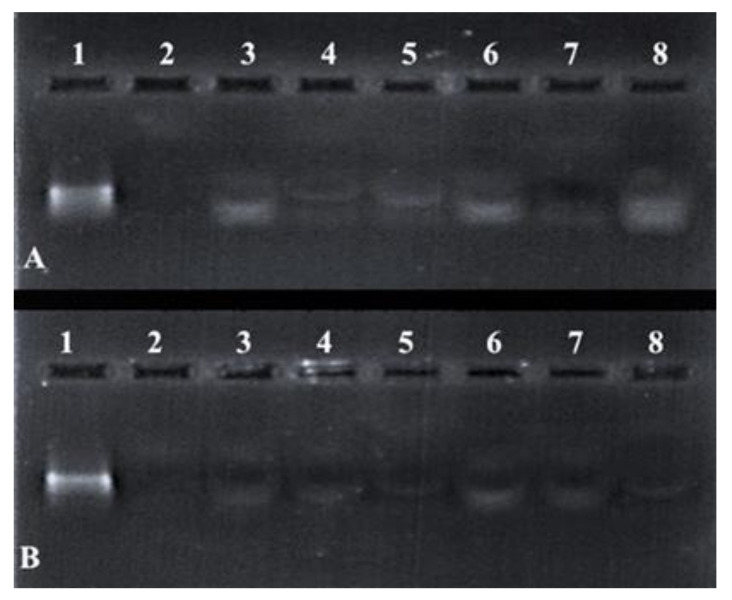
Nuclease protection assay of mRNA: FSeNP nanocomplexes. Lane 1: Positive control (naked mRNA (0.3 µg)). Lane 2: Negative control (mRNA + 10% serum). Nanocomplexes at sub-optimum, optimum, and supra-optimum *^w^/_w_* binding ratios–(**A**) Lanes 3–5: CS-SeNP (1:4.5, 1:5 and 1:5.5) and Lanes 6–8: LA-CS- SeNP (1:5.5, 1:6 and 1:6.5); (**B**) Lanes 3–5: PEG-CS-SeNP (1:4.5; 1:5 and 1:5.5) and Lanes 6–8: PEG-LA-CS-SeNP (1:4.5; 1:5 and 1:5.5).

Differences in the band intensity (Figure 7) of the mRNA indicated that the FSeNPs were capable of protecting the mRNA to varying extents. The bands in Lanes 3–5 and 6–8 (Figure 7A) showed an intense banding pattern compared to those seen for the PEGylated nanocomplexes. Any fluorescence noted in the wells indicated the incomplete release of the mRNA (Figure 7B, Lanes 3–4). The positive control lane (Lane 1) contained naked mRNA with no serum added and showed a distinct band. The negative control (Lane 2) contained mRNA digested by nucleases and showed no visible bands. The overall presence of bands and no smearing confirmed the ability of the FSeNPs to compact and protect the mRNA from nuclease digestion.

### 3.8. Cytotoxicity

**Figure 8:**
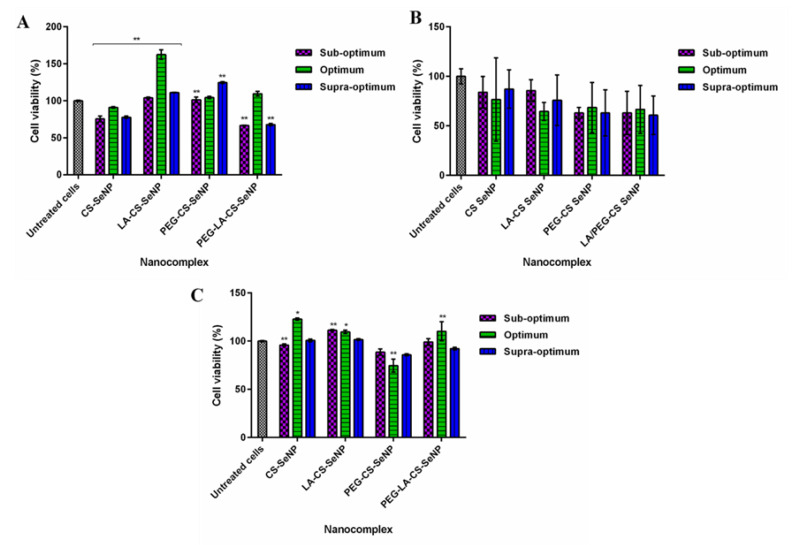
Cytotoxicity profiles of FSeNP nanocomplexes after 48 h in (**A**) HEK293, (**B**) Caco-2, and (**C**) HepG2 cell lines. Column data are represented as the mean ± SD (*n* = 3). Untreated cells were used as the control and taken as 100% viability. * *p* < 0.05 and ** *p* < 0.01 show a statistical significance between similar ratios of targeted vs untargeted nanocomplexes using two-way ANOVA.

Cell viability of >60% was noted for all cell lines treated with the FSeNPs nanocomplexes (Figure 8). No discernible cytotoxicity was observed in the HEK293 and HepG2 cells (Figure 8 A,C) (*p* > 0.05).

The lowest cell viabilities recorded were 62.5% (PEG-LA-CS SeNPs) for HEK293 cells, 60.8% (PEG-LA-CS-SeNP) for the Caco-2 cells, and 88.8% (PEG-CS-SeNPs) for the HepG2 cells. The HepG2 cells were the most tolerant to the nanocomplexes, boding well for their use in targeted gene delivery, with the targeted nanocomplexes exhibiting cell viabilities >100%.

### 3.9. Apoptosis

The majority of the treated cells showed no distinct features corresponding to apoptotic cells with almost all cells appearing green, indicating non-apoptotic cells (Supplementary Figure S3). An early-stage apoptotic cell (yellow) can be seen in the Caco-2 cells treated with the LA-CS-SeNP nanocomplex, and late apoptotic stage cells (orange) can be seen in the HepG2 cells treated with PEG-CS-SeNPs. No dead or necrotic cells were identified. The apoptotic indices were all notably low, ranging from 0.012 to 0.040 (Supplementary Table S1), indicating no significant apoptosis was induced by these FSeNP nanocomplexes. This was in keeping with the low cytotoxicity observed in the MTT assay.

### 3.10. Transfection Assay

**Figure 9:**
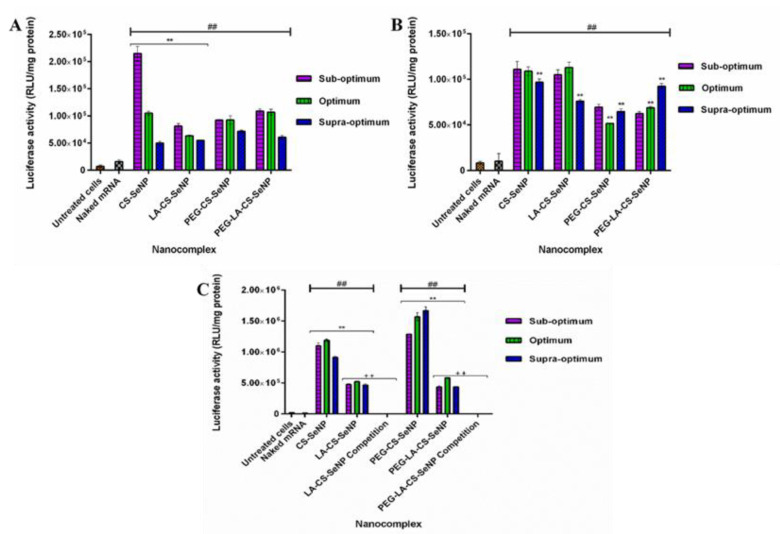
In vitro transfection potential of FSeNP nanocomplexes in the (**A**) HEK293, (**B**) Caco-2, and (**C**) HepG2 cells. An untreated cells control and a naked mRNA control were included. Column data are represented as mean ± SD (*n* = 3): ## *p* < 0.01 showing statistical significance between respective nanocomplexes vs. control 2 (naked mRNA), ** *p* < 0.01 showing a statistical significance between similar ratios of targeted vs untargeted nanocomplexes, ++ *p* < 0.01 showing a statistical significance between similar ratios of targeted vs untargeted nanocomplexes after blocking of the ASGPR. Statistical significance was calculated using two-way ANOVA.

All nanocomplexes were able to successfully deliver and release the mRNA payload in the mammalian cells tested, as evidenced by the significant increase in luciferase activity (*p* < 0.01) observed across all cells compared to the naked mRNA control. Overall, luciferase activity ranged from 5 × 10^4^ to 1.67 × 10^6^ RLU/mg protein. In the HEK293 (Figure 9A) cells, luciferase activity varied from 5 × 10^4^ to 2.15 × 10^5^ RLU/mg protein, while in the Caco-2 cells (Figure 9B), luciferase activity was in the range of 5.17 × 10^4^ to 1.13 × 10^5^ RLU/mg protein. A significant increase in luciferase activity was observed for the targeted HepG2 cells (Figure 9 C) compared to the other two cell lines, with luciferase activity ranging from 4.36 × 10^5^ to 1.67 × 10^6^ RLU/mg protein. These results suggest an increased affinity of the FSeNPs for the HepG2 cells. In the HEK293 cells, maximum luciferase activity of 2.15 × 10^5^ RLU/mg protein was obtained for CS-SeNPs at the sub-optimum ratio, corresponding to a five-fold decrease in luciferase activity compared to that in the HepG2 cells (1.10 × 10^6^ RLU/mg protein). Most FSeNP nanocomplexes showed a concentration-dependent reduction in luciferase activity.

In the Caco-2 cells, a maximum luciferase activity of 1.13 × 10^5^ RLU/mg protein was observed for the LA-CS-SeNP nanocomplexes at the optimum ratio (Figure 9B), which was almost a five-fold reduction to that seen in the HepG2 cells (5.24 × 10^5^ RLU/mg protein). In the HepG2 cells, the PEG-CS-SeNP nanocomplexes showed the highest transfection activity at the supra-optimum ratio (1.67 × 10^6^ RLU/mg protein), while the lowest luciferase activity was noted for PEG-LA-CS-SeNP nanocomplexes (4.36 × 10^5^ RLU/mg protein) at the sub-optimum ratio. Notably, the latter was still higher than that recorded for the HEK293 and Caco-2 cells. However, the transgene expression of the untargeted nanocomplexes was observed to be higher than that of the targeted nanocomplexes in the HepG2 cells.

### 3.11. Receptor Competition Assay

**Figure 10:**
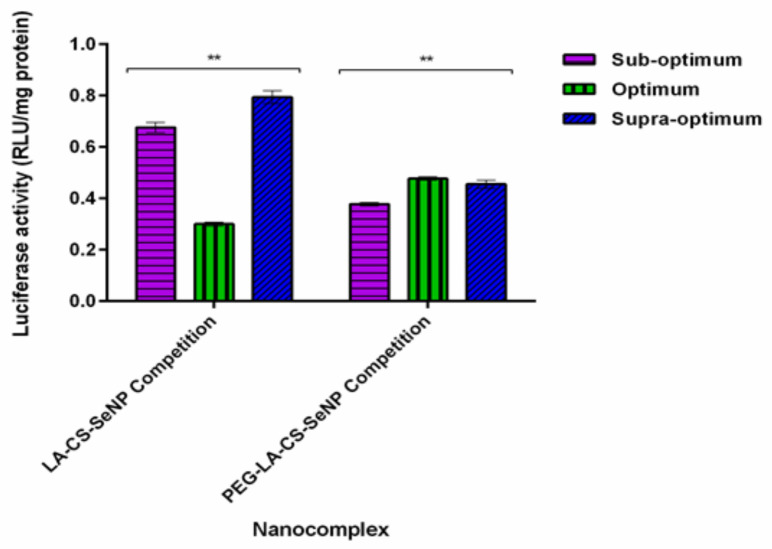
In vitro transfection potential of hepatocellular targeted FSeNPs after blocking of the ASGPR in the receptor competition binding assay in the HepG2 cells. Data are represented as mean ± SD (*n* = 3): ** *p* < 0.01 showing a statistical significance between similar ratios using two-way ANOVA.

The results showed a significant decrease (*p* < 0.01) in luciferase activity in the HepG2 cells treated with the LA-CS-SeNPs and PEG-LA-CS-SeNPs after blocking of the ASGPR at all ratios (Figure 9C and Figure 10). For the LA-CS-SeNPs, luciferase activity ranged from 3 × 10^−1^ to 7.94 × 10^−1^ RLU/mg protein. This was a massive decrease from the maximum luciferase activity of 5.24 × 10^5^ RLU/mg protein observed before blocking of the ASGPR (Figure 9C). Similarly, for the PEG-LA-CS-SeNPs, luciferase activity ranged from 3.78 × 10^−1^ to 4.77 × 10^−1^ RLU/mg protein, corresponding to a considerable decrease from the maximum luciferase activity of 5.83 × 10^5^ RLU/mg protein. This study confirmed that the targeted nanocomplexes were predominantly taken up by receptor-mediated endocytosis.

## 4. Discussion

The reduction of Se species from its Se (IV) oxidative state to its elemental state Se (0) has been shown to reduce selenium’s toxicity and increase bioavailability [45]. The method allowed for control of the size of the SeNP core to around 100 nm in diameter. To increase the stability, as well as enhance the ability of SeNPs to target and deliver the mRNA payload to the hepatocytes of the liver, the surface of SeNPs was modified through functionalization with CS, PEG, and LA, which did see a change in size. Such polymeric coating has been reported to stabilize SeNPs by improving viscosity, reducing inter-particle interaction, and decreasing aggregation [46]. CS facilitated the binding of nucleic acids to the NP via electrostatic interactions between its cationic amine groups and the negatively charged phosphate backbone of the mRNA. The 0.1% (*w*/*v*) CS used was reported to be the optimal concentration for homogeneity and stability of the CS encapsulated SeNPs in solution [36,37,47]. Galactose-based ligands have commonly been used for targeting the asialoorosomucoid glycoprotein receptor (ASGPR) on the surface of hepatocytes [48,49,50,51]. Lactobionic acid (LA) used in this study is a disaccharide composed of galactose units conjugated to gluconic acid. The conjugation of PEG to SeNPs has not been fully explored and was used to provide stability and prevent protein adsorption due to its neutral repeating subunits [52].

UV-visible and FTIR spectroscopy confirmed the synthesis and functionalization of the SeNPs. The PEGylated SeNPs showed less distinct absorption peaks, possibly due to the sharing of electrons with the oxygen of the PEG. This caused the higher orbitals to be occupied, requiring increased energy to oscillate the surface plasmons. Overall, orbital overlapping would lead to a reduction in the bandgap, correlating to the different colors of the NP solutions [42]. Overall, the results from UV-vis spectroscopy are likely due to the combined effects of polymer interactions, aggregation, and size of ligand modified SeNPs. SeNPs were reduced in size upon incorporation of CS, since CS acts a stabilizing agent controlling the size of the NP. This change in size also brought about a change in color as seen in Figure 3. The smaller size obtained for the CS-SeNPs inferred the role of CS as a capping agent that also controlled the aggregation of the SeNPs during synthesis, favoring smaller NPs [46].

Zeta potentials and hydrodynamic sizes that determine the physiological behavior of NPS are essential parameters to consider in nanomedicine [53]. Zeta potentials of ±30 mV, irrespective of the charge, indicate high stability of the NP [54]. Zeta potential can also relate to the ability of the NP to bind and complex mRNA [55]. The high zeta potentials obtained are indicative of good colloidal stability, with the negative charge alluding to the presence of anions on the SeNP surface [39,56,57]. All functionalized SeNPs had an overall positive charge due to the presence of the amines of CS, which orientated outward from the Se core [46,58,59]. The low zeta potential of the LA-CS-SeNPs (16.9 ± 0.7 mV) could be due to the binding of LA to the CS-SeNP. Similar results were reported for folic acid conjugation to ursolic acid CSNPs [60]. NTA sizes were generally smaller than those obtained by TEM, which could be due to the intermolecular and intramolecular forces that hold the NPs together in the solution being negated when the NPs were prepared during TEM, causing the NPs to swell.

The mRNA interaction with the cationic NPs showed good binding and compaction of the mRNA. The band shift assay allowed for the identification of the minimum amount of FSeNPs needed to bind a specific amount of mRNA, which is crucial since high concentrations of SeNPs are known to induce apoptosis and immune responses, while lower concentrations act as a nutritional supplement [61,62,63]. This dose-dependent nature of SeNPs allows for tailoring of dual treatment regimes in which the NP may work synergistically with the genetic payload. Compaction of nucleic acids by NPs plays a critical role in delivery as it protects against nucleases while also facilitating internalization and release of the genetic cargo into the cell [64]. The higher degree of condensation by PEGylated NPs can be attributed to the higher binding potential of these NPs [65]. It can be assumed that single protonated amino groups would interact with multiple phosphate groups at varied points on the mRNA molecule, causing the initial disruption to the mRNA structure displacing the EB and reducing the fluorescence. Furthermore, single-stranded nucleic acids have been reported to interact with the hydrophobic regions of NPs with low zeta potentials, while their interaction with higher zeta potential NPs is mainly through electrostatic forces [55]. The band shift assay allowed us to determine which ratio of NP:mRNA produced optimal mRNA binding. The ratio above (supra-optimal) and ratio below (sub-optimal) were also included in further investigations. All three ratios are important, as sometimes the sub- or supra-optimal ratio provides better transfection than at the optimal ratio, as reported previously [50].

A major problem faced in gene delivery is that nucleases quickly degrade nucleic acids upon entry into the body. mRNA, in particular, is known for its short half-life, and coupled with its inherent instability, it is rapidly degraded by RNases in the body [31,32]. Opsonization by proteins may further hinder the delivery of the payload [66]. The PEGylated NPs showed a reduced ability to protect mRNA compared to the non-PEGylated NPs. PEG is known to provide steric hindrance, stability, and enhanced circulation of the NP in vivo. A similar degradation of mRNA complexed with PEG-Polylysine NPs was seen where the conformation of the PEG molecule around the nucleic acid was unable to protect the mRNA from RNases [67]. The size, density, or intensity of the bands are often not the same as that of the control, which could be due to the tight nanocomplex formed that does not easily release all the mRNA and allow it to run into the gel. Some of the RNA may still be retarded in the gel due to this. The SDS may have been unable to fully disassociate the mRNA from the nanoparticle in these cases. This is something that has been seen especially for inorganic nanoparticles, even when using plasmid DNA [68].

In vivo studies on animal models supplemented with Se or SeNPs revealed that Se commonly accumulates in the liver and kidneys [69,70,71]. From the results obtained, it may be inferred that the HEK293 and HepG2 cells have an ability to maintain viability when exposed to Se, compared to the Caco-2 cells [71], possibly due to their roles in the metabolism and excretion of Se [72]. This may account for the high viability seen in the HepG2 cells. Colorectal cancers have been reported to contain high levels of Se, hence, making them more susceptible to further Se treatment [20,61]. Generally, cancer cells are more vulnerable to the cytotoxic effects of Se compounds at lower concentrations compared to normal cells like the HEK293 [58,61]. For SeNPs, this could occur due to the high levels of reactive oxygen species (ROS) generated in cancer cells as a result of increased metabolism and excess proliferation of cancer cells, known as the Warburg effect [73]. CS encapsulation can reduce SeNP cytotoxicity, enhance stability, and reduce ROS production by controlling the release of Se and its bioavailability to the cells [58,74]. Although Se species were reported to induce cytotoxicity through apoptosis [14,20,61], no significant apoptosis was observed in this study as evidenced in Supplementary Figure S4 and Table S2. Similar results with low apoptotic indices were reported for mRNA delivery using modified SeNPs [36]. Overall, favorable cell viability in normal HEK293 cells bodes well for the use of these NPs in both gene and drug delivery. Similar to chemotherapeutic agents, gene delivery vectors require successful accumulation within the cell to elicit their biological effects. High accumulation of therapeutics in cancer cells can be achieved through the use of passive targeting pathways by nanocarriers. However, this delivery may not specifically target the tumor, thereby limiting its application [75]. The use of mRNA therapeutics further provides a transient high level of expression of the genetic cargo upon entry into the cell, preventing the side effects caused by persistent foreign genetic elements, which could further encourage the transformation of cells into a cancerous state [76]. Transfection capabilities of novel gene vectors are often evaluated by the use of reporter genes (e.g., bioluminescence, β-galactosidase, and fluorescent proteins), which offer a mode of measuring the transcription of foreign genetic elements introduced into the cell through recognition of the accumulation of a produced protein [36,37,38]. The luciferase assay was conducted to assess the transfection potential of these FSeNPs in vitro. Cellular uptake of NPs can be affected by the size, zeta potential, surface modifications, protein corona formation, and cell type [77]. The lower luciferase activity in the Caco-2 cells may be due to their observed reduced tolerance to the nanocomplexes. In the HepG2 cells, the expression of the CS-Se nanocomplexes was higher than that of the targeted nanocomplexes. The large sizes and significantly low zeta potentials of the targeted nanocomplexes may have influenced their overall transfection activities. It is also important to note that the untargeted nanocomplexes possessed zeta potentials closer to –30 mV, correlating to a more stable NP and leading to good luciferase activity, hence, their better transgene expression compared to the targeted nanocomplexes. Nevertheless, the targeted nanocomplexes produced the highest gene expression in the HepG2 cells compared to the other two cell lines, supporting their use as hepatocyte-targeted nanocomplexes.

PEGylation is known to improve the efficacy of delivery vehicles in vivo, which was evident for the luciferase activity obtained in this in vitro study. However, the lower luciferase activity seen for the non-PEGylated NPs in the Caco-2 cells could be due to a lower tolerance of the cells, as mentioned earlier. Despite the negative charge of the CS-Se and PEG-CS-Se nanocomplexes, it was noted that cellular uptake was still favorable, correlating to literature where negatively charged NPs showed reduced protein adsorption and prevention of non-specific cellular uptake [78]. It was reported that internalization of negatively charged NPs is mediated through independent and dependent caveolin and/or clathrin-mediated endocytosis. Internalization of NPs through these pathways is size-dependent, with a size range of 120–150 nm required for internalization and a maximum size of 200 nm being reported for internalization [79]. Although it is accepted that a net positive charge allows for binding to anionic membrane-associated proteoglycans to initiate cellular uptake, it is also possible for mRNA nanocomplexes with a negative zeta potential to enter cells and successfully facilitate gene expression, as we have shown with similar results being reported using siRNA lipoplexes [80]. Hence, the negative zeta potential values obtained do not necessarily imply that these NPs would be unable to associate with the mRNA molecules or the cell membrane. The results from the competition binding assay confirmed that the targeted FSeNPs were taken up primarily through recognition of the ASGPR, which internalized the NPs through clathrin-mediated endocytosis [80]. These findings suggest that the untargeted NPs may have been internalized by the HepG2 cells through both clathrin- and caveolin-mediated endocytosis, which could have led to their high transfection efficiency. PEG-LA-CS-Se nanocomplexes were larger, but Se may have been released from the core of the NP causing Oswald ripening, leading to a reduced size of the NP, which was then readily internalized by the HepG2 cells.

NPs with a size of over 80 nm would be capable of passively accumulating within the liver [81]. However, some in vivo studies have revealed hepatocellular uptake of NPs as large as 227 nm [82]. These NPs must still traverse the sinusoidal fenestrae (50–200 nm) to evade filtration, localize within the space of the Disse, and mediate interaction with the hepatocytes to allow for cellular uptake [1,82]. Most of these nanocomplexes fell within this size range and could have allowed for passive targeting of the hepatocytes. Active targeting, exploiting the use of the ligand cell interactions through chemical conjugation of ligands to nanocarriers [48], allows for the specific recognition of the nanocarrier by the cell surface to improve cell-specific uptake and enhance therapeutic outcomes. Active targeting was confirmed in this study using a receptor competition binding assay. The excess LA added served to block the binding sites of the ASGPR and prevented the targeted nanocomplexes from binding to the receptor, leading to a reduction in the luciferase activity. This assay confirmed that the targeted FSeNPs were recognized and internalized via the ASGPR receptor on the HepG2 cells. Binding of ligands to the ASGPR is affected by the spatial arrangement of the sugar, hydrophobicity of the linker molecule, and density and branching of the sugar [51,81]. Interaction between the amines of CS and the -OH groups of the LA moiety with the SeNP may have orientated LA into an optimal configuration for ASGPR recognition. The quantitative uptake of Se using ICP was reported and showed that not all the Se added was internalized [36,37] by the cells. However, further investigation of these interactions is necessary.

It has been reported that SeNPs explode within the endosome/lysosome due to the highly acidic conditions, facilitating the release of their cargo into the cytoplasm of the cell [82]. Although CS encapsulation could control the release of Se from the core and is able to compact, protect, and mediate the cellular uptake of nucleic acids, it is unable to promote the “proton sponge effect” to facilitate in endosomal rupture and is easily degraded within the lysosomes [83]. Hence, the use of SeNPs may be an important and desirable component of this delivery nano-vehicle. Ultimately, cellular uptake is a complex and highly diverse mechanism in which numerous cellular factors and intrinsic NP properties must be considered. Overall, our results depict that all FSeNP nanocomplexes were able to successfully protect and facilitate the internalization of the mRNA payload into cells, boding well for the use of this mRNA nanosystem in targeted liver cancer immunotherapy.

## 5. Conclusions

SeNPs were successfully synthesized and encapsulated with CS to form CS-SeNPs, to mediate mRNA binding, improve stability, and lower cytotoxicity. Ligand modification with LA and PEG, to constitute hepatocellular targeted LA-CS-SeNPs, stealth PEG-CS-SeNPs, and the dual-targeted stealth PEG-LA-CS-SeNPs, was accomplished. Although the mRNA nanocomplexes possessed negative zeta potentials, this did not hinder their cellular uptake. All FSeNPs favorably bound and protected the mRNA and displayed no significant cytotoxicity or apoptotic induction, auguring well for their in vivo application. Favorable transgene expression in the HepG2 cells was attained by all nanocomplexes, with the LA targeted nanocomplexes being primarily taken up by ASGPR-mediated endocytosis. While this successful delivery of mRNA shows great promise for gene therapy and provides a proof of principle study for future liver-targeted immunotherapy, the multifaceted abilities of mRNA therapy using selenium-based NPs need to be further interrogated. Further studies focusing on improving the physicochemical properties of the FSeNPs, enhancing their stability and mRNA protection, evaluating their intracellular trafficking, Se uptake and release, and cell cycle may help in further understanding the biological effect of these nanocomplexes and their potential as gene nanocarriers. This may unlock the potential to create personalized dual selenium-immunotherapeutic systems, with the possibility of mRNA mediated gene delivery being able to provide long-term gene correction and possibly rival the use of DNA in corrective gene therapy.

## Figures and Tables

**Table 1:** Size distribution, zeta potential and polydispersity indices of all nanoparticles.

Nanoparticle	TEM Size (nm ± SD)	Hydrodynamic Diameter (nm ± SD)	Zeta Potential (mV ± SD)	Polydispersity Index (PDI)
SeNP	101.30 ± 7.43	83.8 ± 0.9	−45.8 ± 0.9	0.0097
CS-SeNP	80.52 ± 17.19	57.2 ± 0.6	20.5 ± 0.1	0.0063
LA-CS-SeNP	113.17 ± 20.30	130.0 ± 4.3	16.9 ± 0.7	0.0932
PEG-CS-SeNP	123.49 ± 31.87	91.4 ± 6.1	31.4 ± 0.6	0.1330
PEG-LA-CS-SeNP	120.25 ± 18.07	85.0 ± 9.3	14.9 ± 0.2	0.1859

**Table 2:** Hydrodynamic size distribution, zeta potential, and polydispersity of mRNA:SeNP nanocomplexes at their optimal binding mRNA:NP *w/w* ratios: (CS-SeNPs (1:5), LA-CS-SeNPs (1:6), PEG-CS-SeNPs (1:5), and LA-PEG-CS-SeNPs (1:5).

Nanocomplex	Hydrodynamic Diameter (nm ± SD)	Zeta Potential (mV ± SD)	Polydispersity Index
mRNA:CS-SeNP	70.6 ± 4.7	−10.4 ± 0.9	0.031
mRNA:LA-CS-SeNP	176.0 ± 61.3	−3.7 ± 2.4	0.120
mRNA:PEG-CS-SeNP	110.2 ± 16.2	−22.5 ± 0.3	0.140
mRNA:PEG-LA-CS-SeNP	263.9 ± 87.5	−4.1 ± 1.2	0.048

## Data Availability

The data and contributions presented in the study are included in the article and Supplementary Material. Further inquiries can be directed to the corresponding author.

## Supplementary Materials

The following are available online at https://www.mdpi.com/1999-4923/13/3/298/s1. Figure S1: FTIR spectrum of (A) CS and (B) CS-SeNP; Figure S2: FTIR spectrum (A) LA-CS-SeNP, (B) PEG-CS-SeNP, and (C) PEG-LA-CS-SeNP; Figure S3: Fluorescent images obtained from dual acridine orange/ethidium bromide apoptosis studies after treatment with FSeNPs in the HEK293, Caco-2, and HepG2 cell lines at 20 × magnification; Table S1: Apoptotic indices of FSeNP nanocomplexes in HEK293, Caco-2, and HepG2 cell lines.
