# Supplementary material for: Hepatocellular-Targeted mRNA Delivery Using Functionalized Selenium Nanoparticles In Vitro
Source: Pharmaceutics. 2021 Feb 24;13(3):298. doi: 10.3390/pharmaceutics13030298 (PMC8025905; doi:10.3390/pharmaceutics13030298)
Supplement: Supplementary file 1 [file pharmaceutics-13-00298-s001.pdf]

pharmaceutics

Supplementary Materials: Hepatocellular-Targeted mRNA Delivery Using Functionalized Selenium Nanoparticles In Vitro

Dhireshan Singh and Moganavelli Singh

Citation: Singh, D.; Singh, M. Hepatocellular-Targeted mRNA Delivery Using Functionalized Selenium Nanoparticles In Vitro. *Pharmaceutics* **2021**, *13*, 298, https://doi.org/10.3390/pharmaceutics13030298

Publisher's Note: MDPI stays neutral with regard to jurisdictional claims in published maps and institutional affiliations.

Copyright: © 2021 by the authors. Submitted for possible open access publication under the terms and conditions of the Creative Commons Attribution (CC BY) license (http://creativecommons.org/licenses/by/4.0/).

1 AUGGAGGACG CCAAGAACAU CAAGAAGGGC CCCGCCCCCU UCUACCCCCU GGAGGACGGC ACCGCCGGCG AGCAGCUGCA CAAGGCCAUG AAGCGGUACG

101 CCCUGGUGCC CGGCACCAUC GCCUUCACCG ACGCCCACAU CGAGGUGGAC AUCACCUACG CCGAGUACUU CGAGAUGAGC GUGCGGCUGG CCGAGGCCAU

201 GAAGCGGUAC GGCCUGAACA CCAACCACCG GAUCGUGGUG UGCAGCGAGA ACAGCCUGCA GUUCUUCAUG CCCGUGCUGG GCGCCCUGUU CAUCGGCGUG

301 GCCGUGGCCC CCGCCAACGA CAUCUACAAC GAGCGGGAGC UGCUGAACAG CAUGGGCAUC AGCCAGCCCA CCGUGGUGUU CGUGAGCAAG AAGGGCCUGC

401 AGAAGAUCCU GAACGUGCAG AAGAAGCUGC CCAUCAUCCA GAAGAUCAUC AUCAUGGACA GCAAGACCGA CUACCAGGGC UUCCAGAGCA UGUACACCUU

501 CGUGACCAGC CACCUGCCCC CCGGCUUCAA CGAGUACGAC UUCGUGCCCG AGAGCUUCGA CCGGGACAAG ACCAUCGCCC UGAUCAUGAA CAGCAGCGGC

601 AGCACCGGCC UGCCCAAGGG CGUGGCCCUG CCCCACCGGA CCGCCUGCGU GCGGUUCAGC CACGCCCGGG ACCCCAUCUU CGGCAACCAG AUCAUCCCCG

701 ACACCGCCAU CCUGAGCGUG GUGCCCUUCC ACCACGGCUU CGGCAUGUUC ACCACCCUGG GCUACCUGAU CUGCGGCUUC CGGGUGGUGC UGAUGUACCG

801 GUUCGAGGAG GAGCUGUUCC UGCGGAGCCU GCAGGACUAC AAGAUCCAGA GCGCCCUGCU GGUGCCCACC CUGUUCAGCU UCUUCGCCAA GAGCACCCUG

901 AUCGACAAGU ACGACCUGAG CAACCUGCAC GAGAUCGCCA GCGGCGGCGC CCCCCUGAGC AAGGAGGUGG GCGAGGCCGU GGCCAAGCGG UUCCACCUGC

1001 CCGGCAUCCG GCAGGGCUAC GGCCUGACCG AGACCACCAG CGCCAUCCUG AUCACCCCCG AGGGCGACGA CAAGCCCGGC GCCGUGGGCA AGGUGGUGCC

1101 CUUCUUCGAG GCCAAGGUGG UGGACCUGGA CACCGGCAAG ACCCUGGGCG UGAACCAGCG GGGCGAGCUG UGCGUGCGGG GCCCCAUGAU CAUGAGCGGC

1201 UACGUGAACA ACCCCGAGGC CACCAACGCC CUGAUCGACA AGGACGGCUG GCUGCACAGC GGCGACAUCG CCUACUGGGA CGAGGACGAG CACUUCUUCA

1301 UCGUGGACCG GCUGAAGAGC CUGAUCAAGU ACAAGGGCUA CCAGGUGGCC CCCGCCGAGC UGGAGAGCAU CCUGCUGCAG CACCCCAACA UCUUCGACGC

1401 CGGCGUGGCC GGCCUGCCCG ACGACGACGC CGGCGAGCUG CCCGCCGCCG UGGUGGUGCU GGAGCACGGC AAGACCAUGA CCGAGAAGGA GAUCGUGGAC

1501 UACGUGGCCA GCCAGGUGAC CACCGCCAAG AAGCUGCGGG GCGGCGUGGU GUUCGUGGAC GAGGUGCCCA AGGGCCUGAC CGGCAAGCUG GACGCCCGGA

1601 AGAUCCGGGA GAUCCUGAUC AAGGCCAAGA AGGGCGGCAA GAUCGCCGUG UGA

Figure S1. Sequence of mRNA used in study—proprietary of Trilink BioTechnologies, Inc (San Diego, CA, USA).

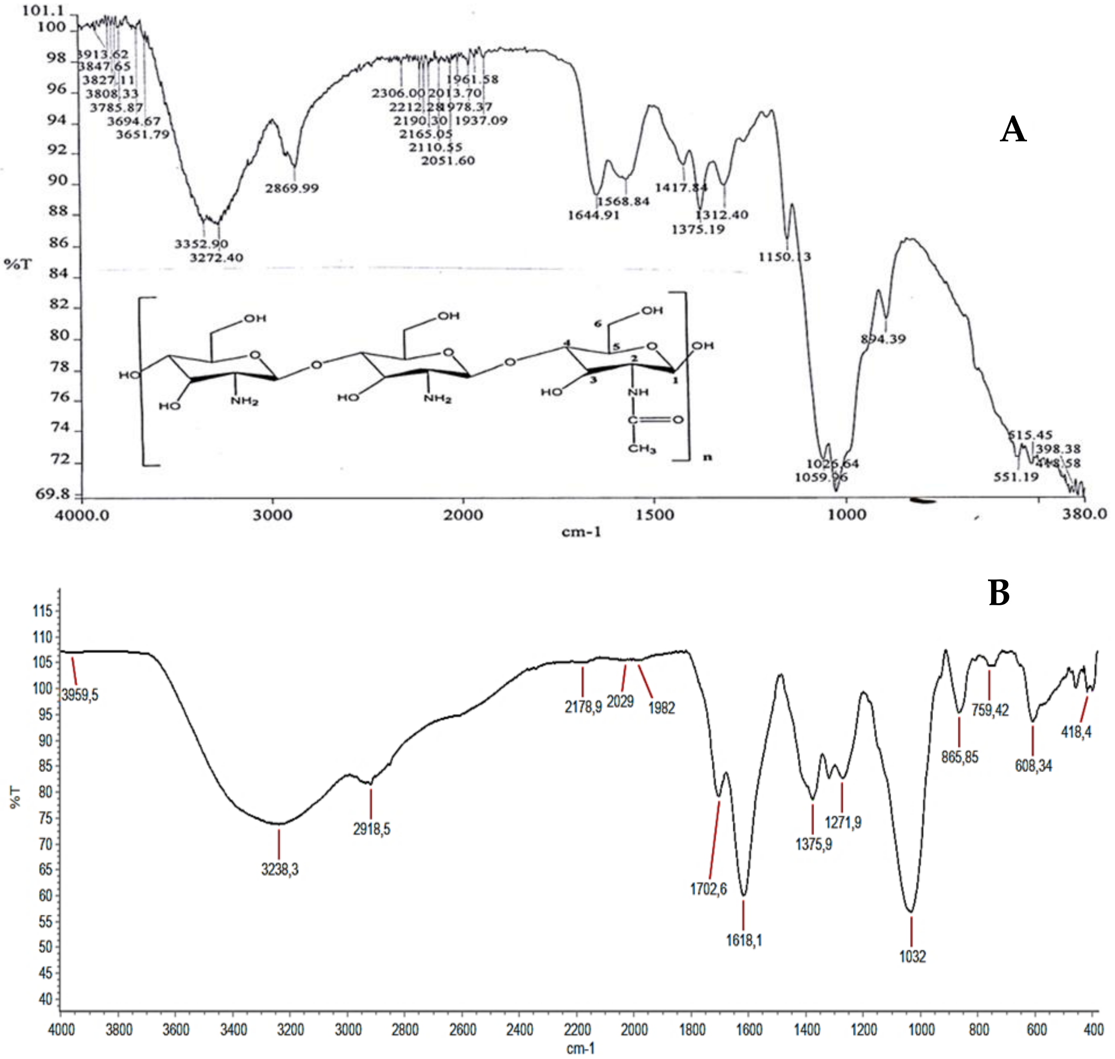

Figure S2. FTIR spectrum of **(A)** CS and **(B)** CS-SeNP.

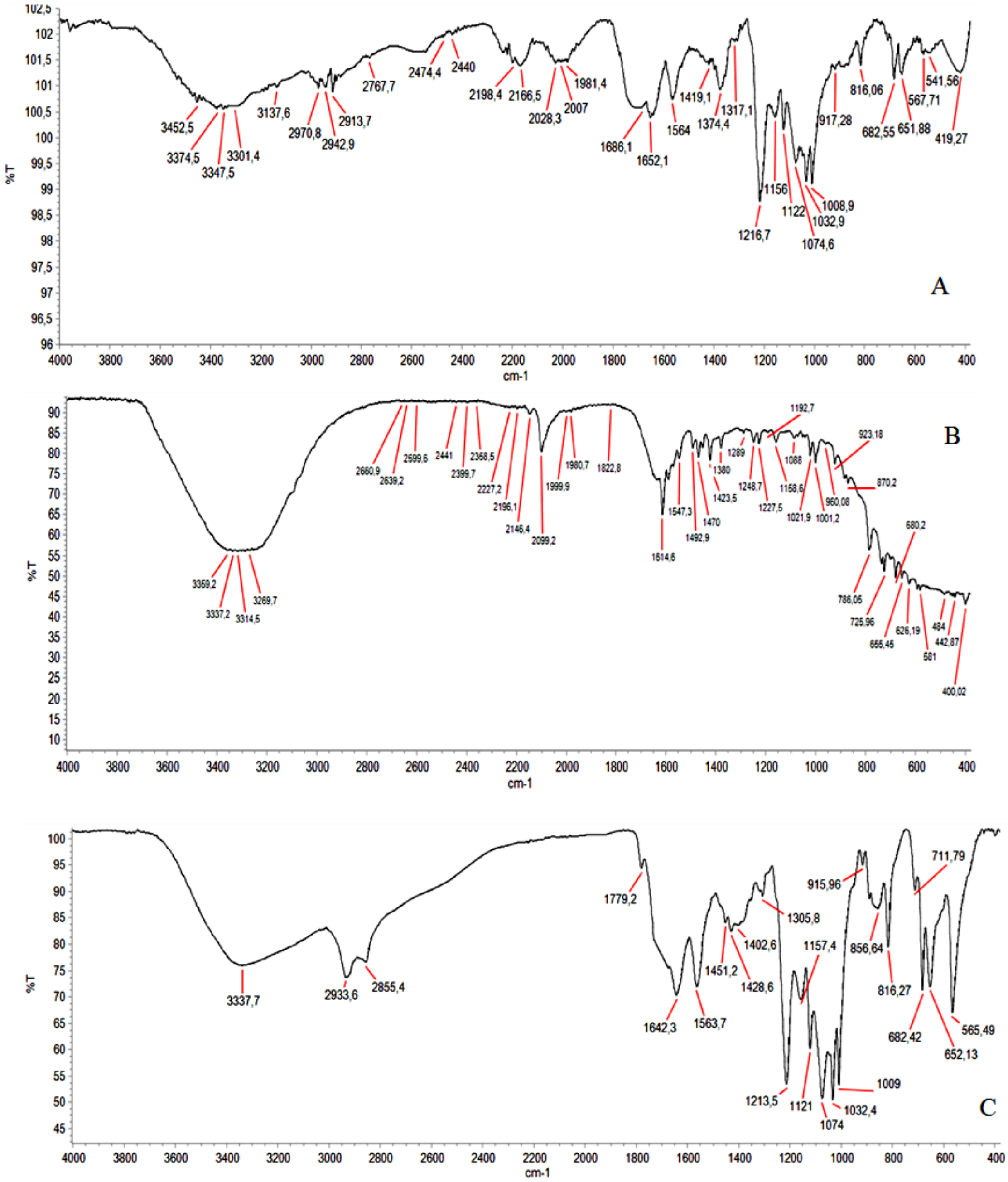

Figure S3. FTIR spectrum **(A)** LA-CS-SeNP, **(B)** PEG-CS-SeNP, and **(C)** PEG-LA-CS-SeNP.

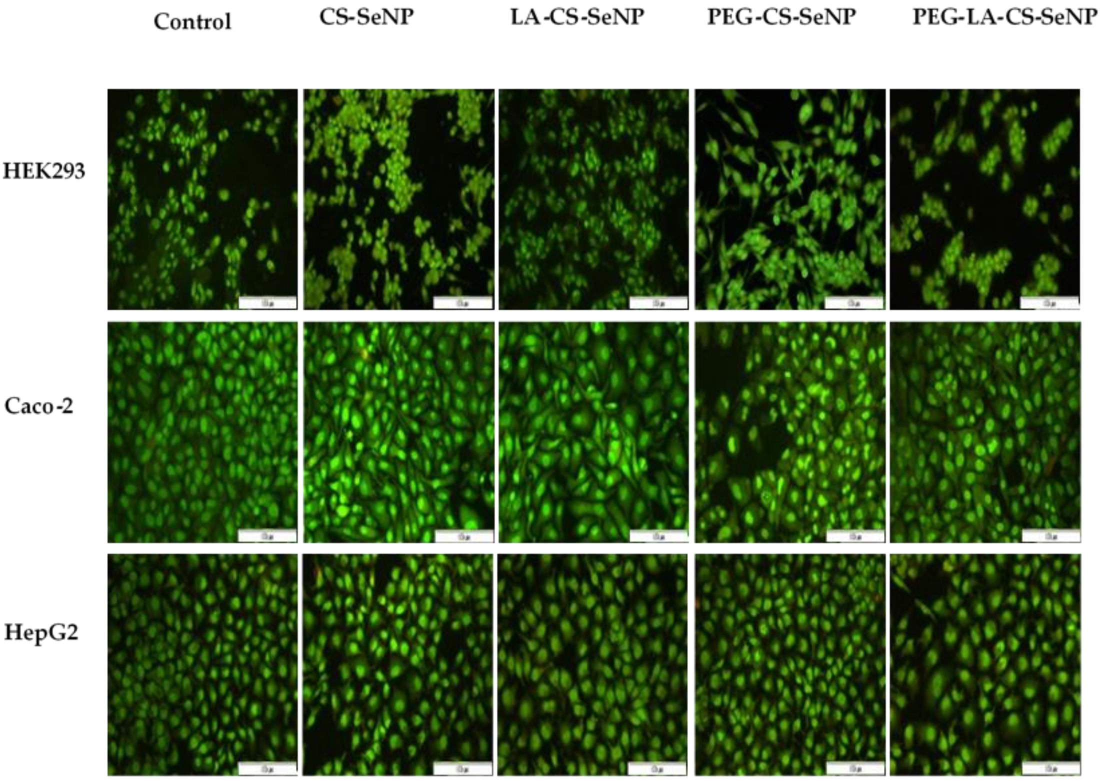

Figure S4. Fluorescent images obtained from dual acridine orange/ethidium bromide apoptosis studies after treatment with FSeNPs in the HEK293, Caco-2 and HepG2 cell lines at 20× magnification.

Table S1. Sub-optimum, optimum, and supra-optimum functionalized SeNP:mRNA (*w*/*w*) ratios identified in the band shift assay.

	**CS-SeNP**		
**Components**	**Ratio**		
	**Sub-optimum**	**Optimum**	**Supra-optimum**
	1:4.5	1:5	1:5.5
mRNA (μg)	0.30	0.30	0.30
Nanoparticle (μg)	1.35	1.51	1.64
	**LA-CS-SeNP**		
Components	**Ratio**		
	**Sub-optimum**	**Optimum**	**Supra-optimum**
	1:5.5	1:6	1:6.5
mRNA (μg)	0.30	0.30	0.30
Nanoparticle (μg)	1.82	1.96	2.10
	**PEG-CS-SeNP**		
Components	**Ratio**		
	**Sub-optimum**	**Optimum**	**Supra-optimum**
	1:4.5	1:5	1:5.5
mRNA (μg)	0.30	0.30	0.30
Nanoparticle (μg)	1.32	1.50	1.65
	**PEG-LA-CS-SeNP**		
Components	**Ratio**		
	**Sub-optimum**	**Optimum**	**Supra-optimum**
	1:4.5	1:5	1:5.5
mRNA (μg)	0.30	0.30	0.30
Nanoparticle (μg)	1.32	1.50	1.65

Table S2. Apoptotic indices of FSeNP nanocomplexes in the HEK293, Caco-2, and HepG2 cell lines.

**Cell line.**	**Apoptotic index**			
	**CS-SeNPs**	**LA-CS-SeNPs**	**PEG-CS-SeNPs**	**LA-PEG-CS-SeNPs**
HEK293	0.033	0.012	0.032	0.040
Caco-2	0.032	0.026	0.039	0.015
HepG2	0.035	0.014	0.021	0.031
